# Supplementary material for: Effects of High Glucose on Human Endothelial Cells Exposed to Simulated Microgravity
Source: Biomolecules. 2023 Jan 17;13(2):189. doi: 10.3390/biom13020189 (PMC9952903; doi:10.3390/biom13020189)
Supplement: Supplementary file 1 [file biomolecules-13-00189-s001.zip › biomolecules-2149553-supplementary.pdf]

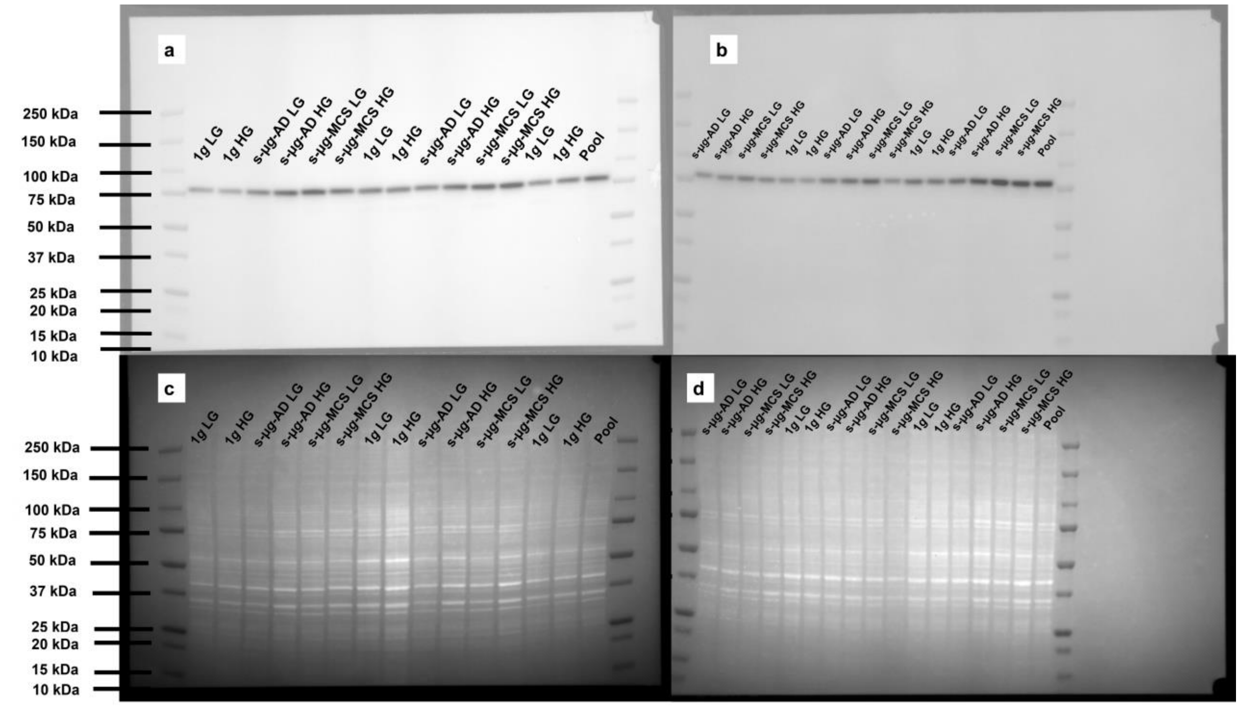

Figure S1. Full-length Western blot images for transglutaminase-2 (TG2): **a**) 1st blot after development with ECL, **b**) 2nd blot after development with ECL, **c**) 1st membrane after transfer for total protein quantification, **d**) 2nd membrane after transfer for total protein quantification. n = 5

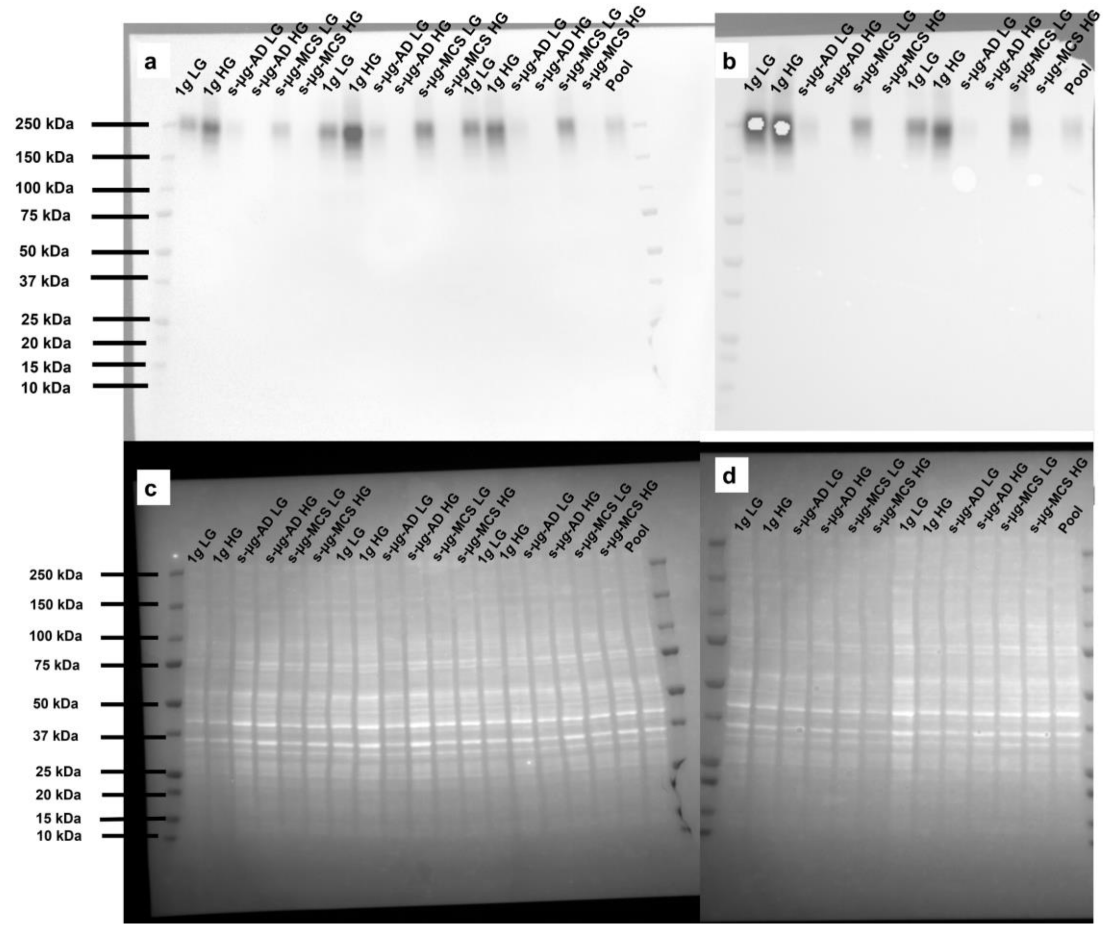

Figure S2. Full-length Western blot images for fibronectin (FN1). **a**) 1st blot after development with ECL, **b**) 2nd blot after development with ECL, **c**) 1st membrane after transfer for total protein quantification, **d**) 2nd membrane after transfer for total protein quantification. n = 5, overexposed 1*g* LG and 1*g* HG samples were excluded.

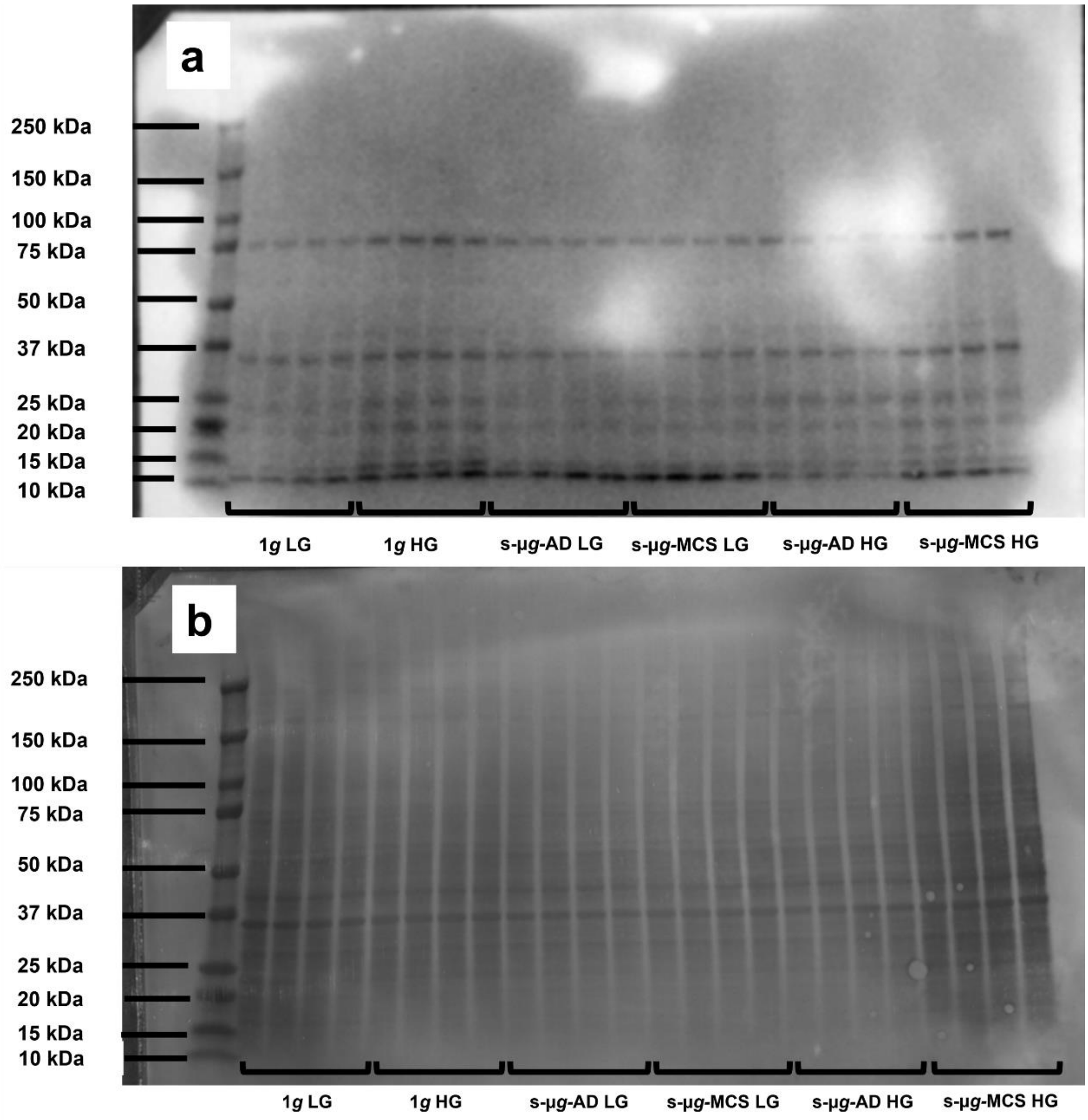

Figure S3. Full-length Western blot images for caspase-3 (CASP3). **a**) Full blot after development with ECL, **b**) Membrane after transfer for total protein quantification. n = 4

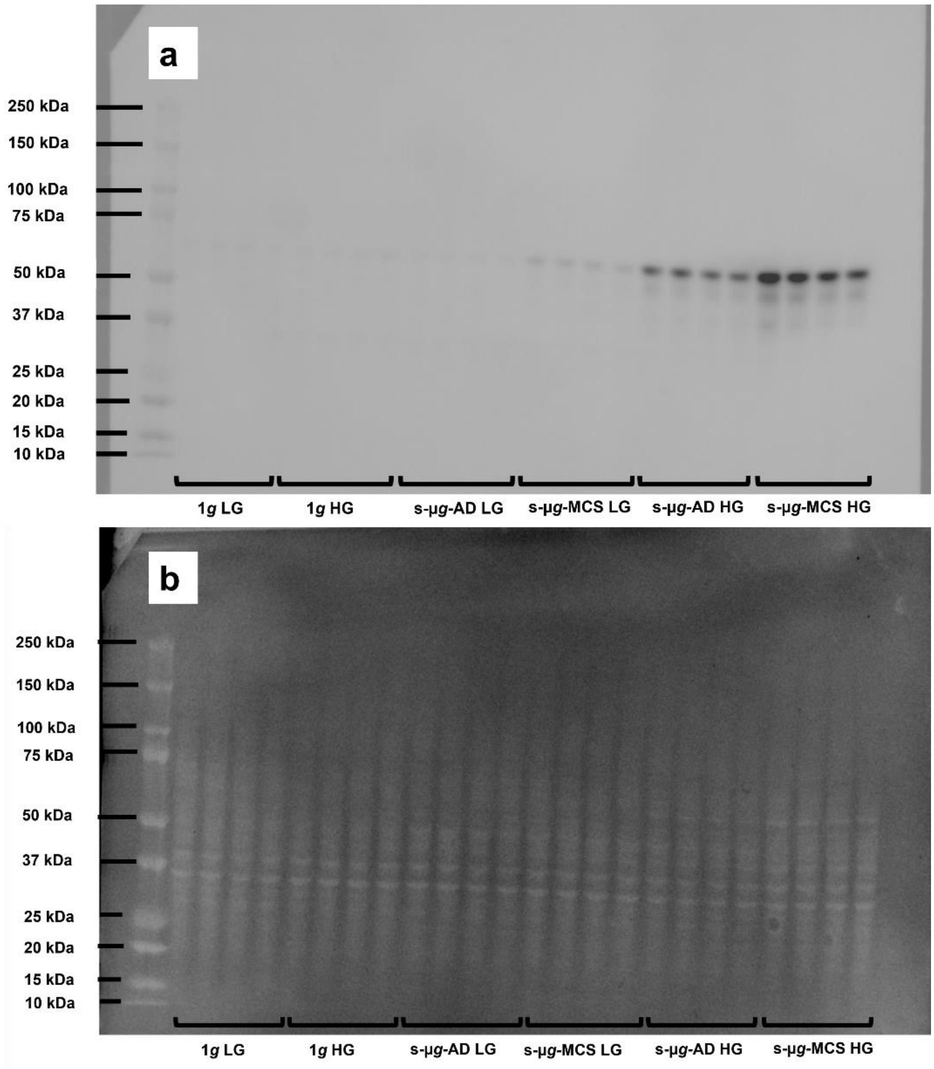

Figure S4. Full-length Western blot images for NF-κB p65. **a**) Full blot after development with ECL, **b**) Membrane after transfer for total protein quantification. n = 4

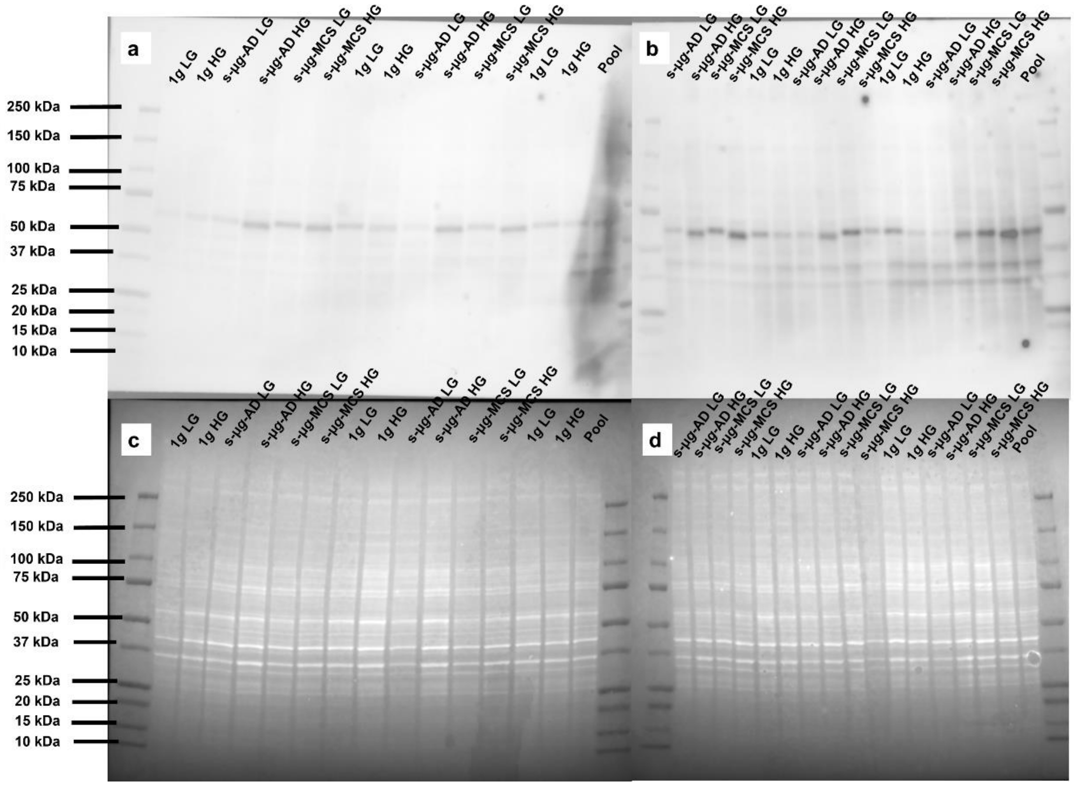

Figure S5. Full-length Western blot images for NADPH oxidase 4 (NOX4). **a**) Full blot after development with ECL, **b**) Membrane after transfer for total protein quantification. n = 5
